# High intake of sweet foods and low life satisfaction can act as risk factors for acute coronary syndrome through synergistic interaction

**DOI:** 10.3389/fnut.2023.1221916

**Published:** 2023-08-07

**Authors:** Jisun So, Kyong-Mee Chung, Jihyeon Seo, Byungmi Kim, Hyejin Chun, Sung Nim Han, Ick-Mo Chung

**Affiliations:** ^1^Department of Food and Nutrition, College of Human Ecology, Seoul National University, Seoul, Republic of Korea; ^2^Department of Psychology, Yonsei University, Seoul, Republic of Korea; ^3^Division of Cancer Prevention, National Cancer Control Institute, National Cancer Center, Goyang-si, Republic of Korea; ^4^Department of Family Medicine, Mokdong Hospital, Ewha Womans University School of Medicine, Seoul, Republic of Korea; ^5^Research Institute of Human Ecology, Seoul National University, Seoul, Republic of Korea; ^6^Division of Cardiology, Mokdong Hospital, Ewha Womans University School of Medicine, Seoul, Republic of Korea

**Keywords:** coronary artery disease, sweets, life satisfaction, diet, psychology

## Abstract

**Purpose:**

Dietary and psychological status contributes to the development of coronary artery disease. However, these lifestyle factors may vary depending on ethnic and environmental background, and secondary prevention programs dealing with these factors in a specific population are not well-established. We aimed to assess dietary and psychological characteristics in Korean patients with acute coronary syndrome (ACS) and analyze their interactions as independent risk factors for ACS.

**Methods:**

Ninety-two patients with ACS (29 acute myocardial infarction and 63 unstable angina) and 69 controls were subjected to dietary and psychological analyses. Dietary intake was assessed by a food frequency questionnaire. Psychological depression and perceived stress were assessed using the Patient Health Questionnaire-9 and the Perceived Stress Scale, respectively. Eight domains of life satisfaction (marital/love relationship, leisure, standard of living, job, health, family life, sex life, and self) were assessed using the Domain Satisfaction Questionnaire (DSQ).

**Results:**

The ACS group had a higher consumption of sweets and fish/seafood, as well as higher levels of depressive symptoms. Additionally, they had lower DSQ scores in total, and all eight individual domains compared with the control group. In multivariate logistic regression analysis, sweet intake (OR 4.57, 95% CI: 1.94–11.40) and total DSQ scores (OR 0.34, 95% CI: 0.14–0.81) were identified as independent risk factors for ACS. Furthermore, these factors, which displayed a significant inverse correlation (ρ = −0.23, *p* = 0.01), were determined as having a synergistic contribution to the development of ACS.

**Conclusion:**

High sweet food intake and low life satisfaction can act as risk factors for ACS through a synergistic interaction, which emphasizes a demand for a more comprehensive approach to secondary prevention of ACS. In addition, these data highlight the role of positive psychological wellbeing factors in cardiovascular health.

## Introduction

The mortality rate of coronary artery diseases (CAD), a top leading cause of death and disease burden globally (1), has notably decreased in recent decades in substantial part due to improved medical management strategies for major modifiable risk factors such as high blood pressure or blood cholesterol levels (2). Along with conventional medical interventions, the adoption of therapeutic lifestyle changes has been acknowledged as the essential basis for managing these risk factors (3, 4). Despite the guidelines that emphasize cardiac rehabilitation on comprehensive lifestyle core components, most programs in the field are under-resourced, especially regarding patient-centered nutritional and psychological counseling (5).

According to the recent systematic analysis conducted on the Global Burden of Disease (GBD) Study, cardiovascular disease (CVD) was revealed as the primary cause of mortality and disability-adjusted life-years attributed to diet (6). In that vein, diet is one of the most studied lifestyle factors for cardiovascular risk, as highlighted in international dietary recommendations such as the dietary approaches to stop hypertension (DASH) (7) and the American Heart Association (AHA) guidelines (8). These guidelines recommend a holistic change in the dietary pattern to have more fruits, vegetables, nuts, legumes, fish, vegetable oil, low-fat dairy, and whole grains and to limit red meats, processed meats, refined grains, added sugars, and salts. However, compliance with the guidelines remains poor in many countries, especially in populations with higher CVD risk (9–11). This indicates a demand for more practical strategies of nutritional care on major food sources that contribute most to cardiovascular risk in a particular population of interest.

Psychological health is another emerging risk and prognostic factor for CAD. The role of psychological maladjustments, such as depression (12), anxiety (13), hostility (14), and stress (15), in developing CAD has been reported. However, as “health” is a complete physical, mental, and social wellbeing and not merely the absence of physical illness (16), psychological wellbeing also needs to be focused on to maintain cardiovascular health (17, 18). Indeed, growing evidence of distinct impacts of psychological maladjustment and wellbeing on cardiovascular biomarkers (19) and future events (20) suggests that both aspects should be well considered in the psychological management of CAD patients.

These lifestyle components can be significantly influenced by ethnic, cultural, and environmental backgrounds, and they can affect each other as well. Cardiac rehabilitation programs that incorporate comprehensive core components have been reported more effective in reducing cardiovascular events (21). For such a comprehensive and strategic intervention program, a careful evaluation of the patient's lifestyle should be preceded. To this end, we assessed the lifestyles of patients with acute coronary syndrome (ACS) by targeting their dietary behaviors and psychological status. We focused on identifying major food items or food groups that are mostly associated with ACS risk, and for psychological status, the indices of both psychological maladjustment and wellbeing were studied. Furthermore, an interactive association of diet and psychological status with the risk of developing ACS was determined.

## Methods

### Study design and participants

Ninety-two patients with ACS and 69 control participants, aged 18–70 years, were recruited and completed the study from January 2012 to March 2013 at Ewha Womans University Mokdong Hospital. The ACS patients had been diagnosed with acute myocardial infarction or unstable angina 2–4 weeks before the analysis. Acute myocardial infarction, which encompasses both non-ST segment elevation myocardial infarction and ST segment elevation myocardial infarction, was diagnosed based on the following three criteria: (1) typical ischemic chest pain, (2) increased cardiac enzyme (hs-cardiac troponin T and CK-MB), and (3) EKG change (ST-T change and pathologic Q wave if present). Unstable angina was diagnosed based on the presence of ischemic chest pain with at least one of the following three features: (1) occurrence at rest, (2) recent onset within the past 2 weeks, and (3) a crescendo pattern characterized by increasing severity, frequency, or duration. Coronary angiography and echocardiography were performed for all patients with ACS. The exclusion criteria included active infection, malignant disease, autoimmune disease, ≥ stage 4 chronic kidney disease (eGFR ≤ 30 ml/min/1.73 m^2^), any other serious medical/surgical illnesses, and a family history of premature CAD or stroke. During the same time period, upon consent, individuals who visited the Health Promotion Center of the hospital for medical check-ups were initially screened for significant CVD or any other medical/surgical conditions based on the record of their annual check-ups. Additionally, participants who were identified to have those conditions through physical examination and laboratory analyses (e.g., EKG, echocardiography, or 3D heart CT angiogram) during the current check-up were subsequently excluded from the study. All participants signed written informed consent, and the study protocol was approved by the Institutional Review Board of Ewha Womans University Mokdong Hospital (ECT 12-01-10).

### Data collection

For the ACS patients, anthropometric information and blood samples were collected during the first or second outpatient visit, which occurred after 1–3 weeks of discharge. Subsequently, on the same day, diet and psychological health assessments were conducted by trained dietitians and psychologists in a separate session by collecting demographic information. For the control participants, all information was collected on the day of their medical check-up visits.

### Diet assessment

Dietary intake was assessed using a food frequency questionnaire (FFQ) during an interview conducted by trained dietitians. The FFQ utilized in this study consists of 103 food items and was developed by the Korea Centers for Disease Control and Prevention specifically for the Korean Genome and Epidemiology Study (KoGES). Its validation was conducted on Korean adults (22, 23). Detailed instructions were given to participants to record food intake estimates over the previous one-year period, especially for patients, for the year preceding their diagnosis. The frequency of consumption was divided into 9 categories: never or seldom, once a month, 2–3 times a month, 1–2 times a week, 3–4 times a week, 5–6 times a week, once a day, twice a day, and three times or more per day. Portions were classified as small, medium, and large, according to the median value of each food consumption from the Korea National Health and Nutrition Examination Survey (KNHANES).

To estimate the nutritional composition of the diet and the frequency of food intake, all FFQs were analyzed by the same dietitians using KoGES software and Can-Pro 4.0 (Korean Nutrition Society, Korea) based on the KoGES database. Can-Pro 4.0 was used to generate data on more nutrients and conduct flexible food grouping. The consistency between the data generated by these two tools was confirmed (data not shown). Each food item, except for 8 items, was classified into one of the main food group categories: rice and grains, starch and starchy vegetables, noodles, instant ramen, meat and poultry, fish and seafood, salted fish, eggs, legumes, nuts, fruits, vegetables, salted vegetables, seaweeds, dairy, sweets, and fast foods (Supplementary Table 1).

### Psychological health assessment

The Patient Health Questionnaire 9 (PHQ-9) was used to assess the severity of depressive symptoms based on the Diagnostic and Statistical Manual of Mental Disorders Fourth Edition (DSM-IV) criteria (24). Participants were asked to rate 9 depressive symptoms over the past 2 weeks using a 4-point scale. The total score ranges from 0 to 27 points with a higher score indicating a higher level of depression (clinical depression: ≥10 points). Cronbach's alpha is 0.83 in the current study.

To measure the level of perceived stress in daily life, we used a modified Korean version of the Perceived Stress Scale (K-PSS), a 10 item-scale, which was developed from the original version with 14 items (25). Participants were asked to rate the level of experienced stress during the previous month based on a five-point scale. An overall score ranges from 0 to 40 points, and a higher score indicates a higher level of stress. Cronbach's alpha was 0.74 in this study.

Life satisfaction was assessed by the Domain Satisfaction Questionnaire (DSQ) which measures eight domains of satisfaction in life: marital/love relationship, leisure, standard of living, job, health, family life, sex life, and self (26). The level of satisfaction in each life domain was rated using a seven-point Likert scale ranging from 1 (very dissatisfied) to 7 (very satisfied). Satisfaction in health was excluded from the analysis as it can be confounded by the diagnosis of ACS in patients. Cronbach's alpha was 0.92 in the present study.

### Assessment of other socioeconomic and lifestyle information

Socioeconomic information, including education, household income, and marital status, as well as other lifestyle components, such as smoking, physical activity, and alcohol consumption, was assessed. Smoking status was determined by smoking experience and daily smoking frequency, resulting in the categorization into 1 of the 4 groups: never smoker, former smoker, and current smoker with <1 pack per day or 1 or more packs per day. The level of alcohol consumption was assessed based on the weekly frequency and the quantity consumed per occasion. Heavy alcohol consumption was defined as both drinking at least twice per week and consuming five or more glasses of alcohol on a single occasion. The assessment of physical activity level took into account the frequency of exercise sessions that are longer than 30 min in duration: light (1–2 sessions per week), moderate (3–5 sessions per week), and heavy (6–7 sessions per week). Low-intensity walking was considered as half a session.

Anthropometric data including waist circumference and body mass index (BMI) were measured. Serum concentrations of glucose, LDL cholesterol, HDL cholesterol, and triglycerides were measured after 12 h of fasting.

### Statistical analysis

Data were presented as means ± SDs or geometric means ± interquartile range, and all analyses were conducted in R version 4.1.0 (27). In a comparison of variables between ACS patients and controls, generalized linear regression models or logistic regression models were used with adjustment for relevant risk factors: sex, age, total energy intake for diet-related variables, and total household income for psychological variables. Multivariate logistic regression models were used to determine independent dietary or psychological risk factors for the development of ACS with adjustment for sex, age, smoking, and/or total energy intake. To explore the interplay between sweet food consumption and total DSQ scores in relation to the risk of ACS, we initially categorized the participants into four groups based on their sweet food intake and DSQ scores, relative to the median values: “Sweets (low) × DSQ (high),” “Sweets (low) × DSQ (low),” “Sweets (high) × DSQ (high),” and “Sweets (high) × DSQ (low)”. Following that, the risk of ACS development was assessed in comparison to the “Sweets (low) × DSQ (high)” group through a multivariate logistic regression analysis while controlling for sex, age, smoking, and/or total energy intake. A linear relationship between daily sweet intake and total DSQ scores was assessed using the Spearman correlation coefficient.

## Results

### Demographic data of subjects

Comparisons of the general characteristics between the ACS patients and controls are presented in Table 1. Compared with the controls, the ACS patients (29 with acute myocardial infarction and 63 with unstable angina) were older, composed of more men, and had heavier smoking habits with lower physical activities. Socioeconomically, the ACS patients belonged to lower household income categories (Supplementary Table 2). The ACS patients tended to have higher BMI and higher waist circumference only in men. The proportion of participants with CAD risk factors, such as dyslipidemia, diabetes, or metabolic syndrome, was higher in the ACS group than controls. Medications taken by the ACS group are shown in Supplementary Table 3.

**Table 1:** General characteristics of study participants.

**Variable**	**ACS (*n* = 92)**	**Control (*n* = 69)**	***p*–value^a^**
**ACS subtype (%)**			
Acute myocardial infarction	29 (31.5)	–	**NA**
Unstable angina	63 (68.5)	–	
Men (%)	73 (79.3)	42 (60.9)	**0.02**
Age, year	53.2 ± 10.2	48.7 ± 6.7	**0.002**
**Smoking (%)**			
Never	21 (22.8)	29 (42.0)	**0.005**
Former	24 (26.1)	11 (15.9)	
Current (<1 pack/day)	14 (15.2)	7 (10.1)	
Current (≥1 pack/day)	32 (34.8)	11 (15.9)	
No response	1 (1.1)	11 (15.9)	
**Alcohol consumption**^b^ **(%)**			
None	32 (34.8)	16 (23.2)	0.08
Moderate	28 (30.4)	30 (43.5)	
Heavy	31 (33.7)	21 (30.4)	
No response	1 (1.1)	2 (2.9)	
**Physical activity**^c^ **(%)**			
None	59 (64.1)	30 (43.5)	**0.02**
Light	17 (18.5)	10 (14.5)	
Moderate	11 (12.0)	18 (26.1)	
Intense	4 (4.3)	9 (13.0)	
No response	1 (1.1)	2 (2.9)	
**Waist circumference, cm**			
Males	88.4 ± 8.4	84.0 ± 9.1	**0.003**
Females	83.0 ± 10.6	78.6 ± 8.6	0.80
Body mass index, kg/m^2^	24.7 ± 3.5	23.8 ± 3.3	0.05
Hypertension^d^ (%)	32 (34.8)	4 (5.8)	0.07
Dyslipidemia^e^ (%)	39 (42.4)	12 (17.4)	**0.003**
Diabetes^f^ (%)	28 (30.4)	3 (4.3)	**0.001**
Metabolic syndrome^g^ (%)	30 (32.6)	3 (4.3)	**<0.001**
Systolic blood pressure, mmHg	125 ± 15	121 ± 12	0.13
Diastolic blood pressure, mmHg	76 ± 9	73 ± 8	0.06
Triglycerides^†^, mg/dl	116 ± 90	91 ± 75	**0.02**
Total cholesterol, mg/dl	179 ± 40	177 ± 26	0.67
HDL cholesterol, mg/dl	44 ± 11	51 ± 11	**<0.001**
LDL cholesterol, mg/dl	113 ± 40	109 ± 26	0.61
Fasting glucose, mg/dl	114 ± 48	89 ± 9	**<0.001**

Values are presented as means ± SDs or ^†^geometric mean ± interquartile range. *n* = 161. Participants who completed either dietary or psychological assessments were included.

ACS, acute coronary syndrome; HDL-C, high-density lipoprotein; LDL, low-density lipoprotein.

^a^*p* values for the comparison between ACS and control groups, except for sex and age variables, were determined using generalized linear models with adjustment for sex and age.

^b^Heavy alcohol consumption is defined as ≥twice per week and ≥5 glasses at once.

^c^Based on the frequency of exercise per week. Exercise longer than ≥ 30 min counted only. Walking is counted as 0.5. Light = 1–2/week; moderate = 3–5/week; heavy = 6–7/week.

^d^Blood pressure ≥140/90 mmHg or use of antihypertensive drugs.

^e^Adult Treatment Panel (ATP) III of the National Cholesterol Education Program (NCEP) (1); total cholesterol ≥240 mg/dl, LDL cholesterol ≥160 mg/dl, triglycerides ≥200 mg/dl or HDL cholesterol <40 mg/dl.

^f^Fasting blood glucose ≥126 mg/dl, HbA1c ≥6.5, or use of antidiabetics.

^g^Three of the following five criteria were met: waist circumference ≥90 cm (men) or 85 cm (women), triglycerides ≥150 mg/dl, HDL cholesterol <40 mg/dl (men) or 50 mg/dl (women), blood pressure ≥140/90 mmHg, and fasting blood glucose ≥100 mg/dl.

*p*-value in bold indicates *p*-value <0.05.

### Comparison of dietary intakes

The nutrient and food group intakes were compared between the ACS patients and the controls, separated by sex (Table 2) or without separation of sex (Supplementary Table 4). The total energy intake of the ACS patients was significantly higher than that of the controls in men but not in women (Table 2a). With age and total energy intake adjusted, men in the ACS group consumed 22% more selenium, and women in the ACS group consumed 54% more vitamin D and 40% more vitamin B12 than in the controls over the past 1 year (Table 2b). Since the content of these nutrients is typically high in foods of animal origin, we analyzed animal-source protein and fat intakes but found no significant inter-group difference in both men and women (data not shown).

**Table 2:** Nutrient and food group intakes in male and female ACS patients and controls.

**Variable**	**Men**			**Women**		

	**ACS (*****n*** = **72)**	**Control (*****n*** = **38)**	***p*** **-value**	**ACS (*****n*** = **19)**	**Control (*****n*** = **23)**	***p*** **-value**
**a) Energy intakes** ^†^ **, kcal**	2,083 ± 523	1,863 ± 565	**0.03**	1,998 ± 451	1,842 ± 563	0.89
**b) Nutrient intakes**						
**Macronutrients**						
Carbohydrate, g	374 ± 93	337 ± 101	0.78	371 ± 83	334 ± 110	0.31
Dietary fiber, g	19 ± 6	18 ± 8	0.57	22± 5	22 ± 10	0.06
Fat, g	39 ± 17	35 ± 17	0.78	32 ± 22	33 ± 15	0.40
Cholesterol, mg	250 ± 129	202 ± 142	0.22	206 ± 128	212 ± 141	0.86
SFA, g	9.9 ± 5.2	8.4 ± 4.3	0.33	8.5 ± 6.7	8.6 ± 5.4	0.53
MUFA, g	10.5 ± 5.7	8.5 ± 4.0	0.21	9.2 ± 7.8	9.8 ± 6.5	0.86
PUFA, g	5.4 ± 2.9	4.0 ± 1.6	0.05	4.4 ± 2.9	4.5 ± 2.3	0.61
Protein, g	78 ± 24	67 ± 23	0.36	74 ± 25	70 ± 25	0.50
**Fat-soluble vitamins**						
Vitamin A, μg RAE	547 ± 264	562 ± 318	0.27	646± 285	672 ± 429	0.23
Vitamin D, μg	4.4 ± 3.8	3.1 ± 2.0	0.17	4.0 ± 2.6	2.6 ± 1.5	**0.003**
Vitamin E, mg α-TE	9.5 ± 3.9	7.9 ± 3.2	0.47	8.9 ± 2.6	8.7 ± 3.2	0.20
Vitamin K, μg	178 ± 137	146 ± 111	0.53	205 ± 92	219 ± 185	0.53
**Water-soluble vitamins**						
Vitamin C, mg	80 ± 44	81 ± 49	0.41	106 ± 36	112 ± 72	0.09
Niacin, mg NE	17 ± 6	15 ± 5	0.51	17 ± 6	16 ± 7	0.92
Vitamin B6, mg	1.6 ± 0.4	1.4 ± 0.5	0.88	1.7 ± 0.4	1.6 ± 0.6	0.46
Folate, μg DFE	481 ± 167	427 ± 193	0.79	533 ± 137	519± 209	0.28
Vitamin B12, μg	7.6 ± 4.2	5.5 ± 3.2	0.10	8.0 ± 5.1	5.7 ± 3.4	**0.02**
**Minerals**						
Calcium, mg	466 ± 194	425 ± 228	0.84	489 ± 135	451 ± 214	0.92
Phosphorus, mg	1,061 ± 327	907 ± 338	0.38	1,099 ± 278	990 ± 355	0.42
Sodium, mg	3,001 ± 1,269	2,617 ± 1,548	0.42	2,645 ± 1,229	2,446 ± 1,158	0.86
Iron, mg	14 ± 5	12 ± 4	0.87	15 ± 3	14 ± 6	0.41
Selenium, μg	118 ± 34	97 ± 32	**0.01**	106 ± 36	97 ± 32	0.28
**c) Food group intakes, serving** ^‡^						
Rice and grains	2.90 ± 0.83	2.60 ± 0.93	0.61	2.65 ± 0.80	2.26 ± 0.99	0.92
Starch and starchy vegetables	0.46 ± 0.49	0.34 ± 0.42	0.36	0.92 ± 1.23	0.54 ± 0.47	0.48
Noodles	0.22 ± 0.23	0.27 ± 0.37	0.08	0.15 ± 0.30	0.18 ± 0.20	0.18
Instant ramen	0.18 ± 0.20	0.15 ± 0.24	0.65	0.06 ± 0.12	0.08 ± 0.07	0.30
Meat and poultry	0.89 ± 0.66	0.84 ± 0.66	0.81	0.65 ± 0.85	0.76 ± 0.69	0.60
Fish and seafood	1.69 ± 1.19	1.05 ± 0.65	**0.03**	2.28 ± 1.70	1.27 ± 0.83	**0.008**
Salted fish	0.06 ± 0.14	0.04 ± 0.05	0.21	0.04 ± 0.07	0.04 ± 0.06	0.78
Eggs	0.33 ± 0.34	0.29 ± 0.46	0.61	0.21 ± 0.24	0.31 ± 0.40	0.32
Legumes	1.00 ± 1.12	0.73 ± 0.62	0.64	0.72 ± 0.47	1.06 ± 0.78	**0.01**
Nuts	0.13 ± 0.26	0.09 ± 0.16	0.98	0.22 ± 0.39	0.23 ± 0.31	0.57
Fruits	1.18 ± 1.24	1.48 ± 1.70	0.10	1.84 ± 1.53	2.02 ± 2.14	0.27
Vegetables	3.11 ± 2.38	2.51 ± 2.24	0.53	3.77 ± 2.12	4.87 ± 3.50	0.07
Salted vegetables	2.98 ± 1.98	2.51 ± 2.07	0.36	2.86 ± 1.99	2.69 ± 1.67	0.75
Seaweeds	0.56 ± 0.42	0.50 ± 0.60	0.77	0.79 ± 0.49	0.56 ± 0.65	0.06
Dairy	0.82 ± 0.91	0.93 ± 1.23	0.36	0.59 ± 0.54	0.57 ± 0.51	0.86
Sweets	2.95 ± 2.01	1.55 ± 2.67	**0.005**	1.50 ± 1.41	0.56 ± 0.43	**0.02**
Fast foods	0.06 ± 0.14	0.04 ± 0.05	0.28	0.01 ± 0.01	0.03 ± 0.03	0.08

Values are means ± SDs. *n* = 152. Participants who completed dietary assessment were included in this analysis. *p*-values for the comparison between ACS and control groups were determined using generalized linear models with adjustment for age and total energy intake.

^†^*p* value adjusted only for age.

^‡^See Supplementary Table 1 for food grouping details.

*p*-value in bold indicates *p*-value <0.05.

Next, we compared food intakes between the two groups by categorizing the food items into 17 different food groups and estimating the total daily servings of each food group (Table 2c). This was calculated based on the serving sizes of food items from the KoGES FFQ database. The most notable differences in both men and women were higher intake of sweets and fish/seafood in the ACS patients than in the controls (Table 2c). Among the food items analyzed, the most notable differences between the groups were observed in the consumption of added sugars in coffee/tea, specifically sugars added in coffee mixes, under the sweets category, and mackerel in the fish/seafood category (Supplementary Table 5). The women ACS patients consumed significantly less legumes than the controls (Table 2c).

### Comparison of psychological status

While no difference was observed between the groups in perceived stress levels estimated using the K-PSS scores, the levels of depressive symptoms measured using the PHQ-9 scores were significantly higher in the patients than in the controls (Table 3). There was no significant difference in clinical depression (PHQ-9 score ≥10) between the ACS patients and the controls (15.3 vs. 8.1%). The total DSQ score, which refers to the average life satisfaction scores across the 8 life domains, was significantly lower in the patients than in the controls, with the lower DSQ scores in all 8 individual domains (marital/love relationship, leisure, standard of living, job, health, family life, sex life, and self; Table 3). These between-group differences remained even after additional adjustment for total household income.

**Table 3:** Psychological characteristics of ACS patients and controls.

**Variable**	**ACS (*n* = 85)**	**Control (*n* =63)**	***p*** **-value**	

			**Model 1** ^a^	**Model 2** ^b^
Patient health questionnaire-9 (PHQ-9)	5.4 ± 4.7	4.2 ± 3.6	**0.04**	0.07
Perceived stress scale (PSS)	17.4 ± 4.4	17.0 ± 4.1	0.50	0.88
**Domain satisfaction questionnaire (DSQ)**				
Marital/love relationship	4.37 ± 1.41	4.73 ± 1.32	**0.02**	**0.045**
Leisure	4.22 ± 1.26	4.81 ± 1.12	**0.006**	**0.045**
Standard of living	4.07 ± 1.05	4.80 ± 1.06	**<0.001**	**0.003**
Job	4.20 ± 1.08	4.60 ± 1.16	**0.02**	**0.04**
Health	3.98 ± 1.13	4.59 ± 0.92	**<0.001**	**0.005**
Family life	4.35 ± 1.27	4.84 ± 1.14	**0.004**	**0.03**
Sex life	3.73 ± 1.25	4.60 ± 0.97	**<0.001**	**<0.001**
Self	4.04 ± 1.17	4.72 ± 1.10	**<0.001**	**0.001**
Average	4.16 ± 0.90	4.69 ± 0.96	**<0.001**	**0.001**

Values are means ± SDs. *n* = 148. Participants who completed psychological assessment were included in this analysis.

^a^*p*-values for the comparison between ACS and control groups were determined using generalized linear models with adjustment for sex and age.

^b^*p*-values were additionally adjusted for total household income.

*p*-value in bold indicates *p*-value <0.05.

### Associations of dietary and psychological factors to ACS development

In this cohort, we determined the risk of developing ACS associated with each of the aforementioned dietary and psychological factors using multivariate logistic regression models with adjustment for sex, age, and smoking. Especially for estimating the risk related to each food group intake, the models were tested with an additional adjustment for total energy intake. Among the food groups that showed significant differences between the groups, only sweet intake had a significant association with ACS risk with an OR of 4.57 (95% CI 1.94–11.40; Table 4a). However, no significant association was observed between fish/seafood intake and ACS risk. Regarding psychological measures, only the total DSQ score showed a significant inverse association with the risk of ACS with an OR of 0.34 (95% CI 0.14–0.81; Table 4b).

**Table 4:** Odd ratios of ACS associated with select lifestyle factors.

**Variable**	**OR**	**95% Cl**	***p*-value**
**a) Dietary factors**			
Sweet intake (high vs. low)^a^	4.57	(1.94–11.40)	**<0.001**
Fish/seafood intake (high vs. low)^b^	2.18	(0.92–5.34)	0.08
**b) Psychological factors**			
PHQ-9 score (high vs. low)^c^	2.08	(0.89–5.01)	0.10
Total DSQ score (high vs. low)^d^	0.34	(0.14–0.81)	**0.02**

The odds ratios were determined using logistic regression model adjusted for sex, age, and smoking, and for dietary factors, additionally adjusted for total energy intake. *n* = 142 for dietary factors and 118 for psychological factors. CI, confidence interval; DSQ, domain satisfaction score; OR, odds ratio; and PHQ-9, patient health questionnaire 9.

^a^Divided by the median intake of 1.52 daily servings. high (serving >1.52, *n* = 70); low (serving ≤ 1.52, *n* = 72).

^b^Divided by the median intake of 1.3 daily servings. high (serving >1.3, *n* = 71); low (serving ≤ 1.6, *n* = 71).

^c^Divided by the median score of 4. high (score ≥4, *n* = 59); low (score <4, *n* = 59).

^d^Divided by the median score of 4.43. high (score ≥4.43, *n* = 60); low (score <4.43, *n* = 58).

*p*-value in bold indicates *p*-value <0.05.

**Figure 1:**
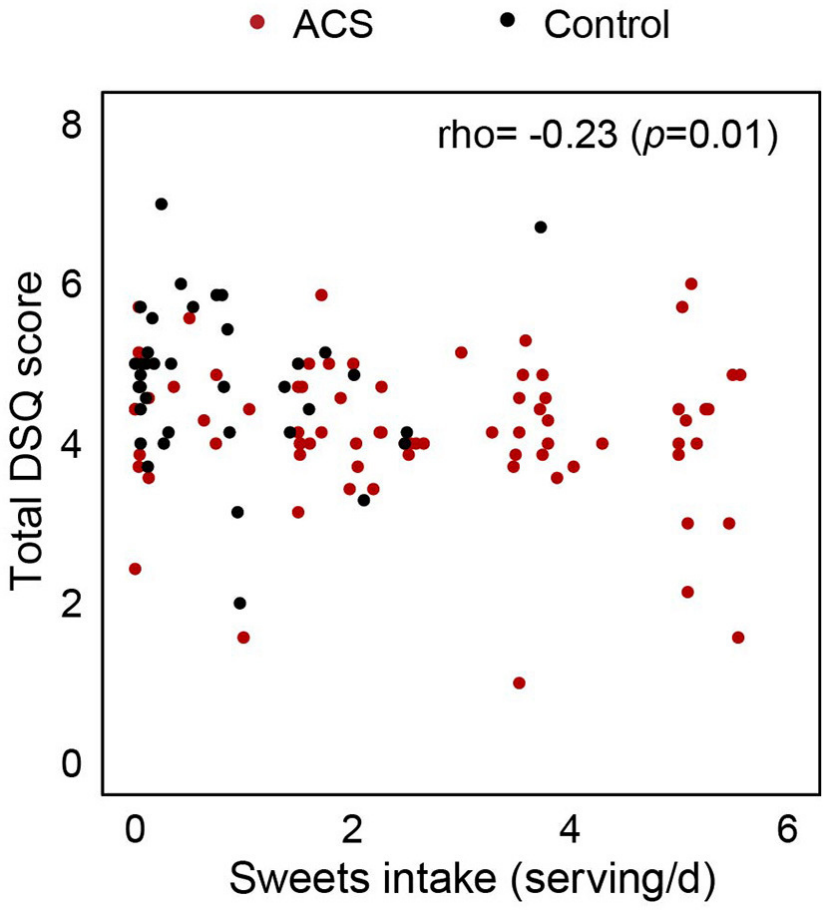
A correlation between daily sweet intake and total domain satisfaction score. The correlation was determined using the Spearman rank correlation coefficient (rho, ρ). Each point represents individual participants: ACS (red) and controls (black). The correlation coefficient and its *p*-value are presented in the top right corner.

Next, we examined an interactive association between sweet food consumption and total DSQ score on the risk of ACS. First, a modest but significant inverse correlation between daily sweet intake and total DSQ score was observed (ρ = −0.23, *p* = 0.01, Figure 1), indicating that those with lower life satisfaction tend to consume more sweet foods or vice versa. In addition, a multivariate logistic regression analysis revealed significantly incremental ACS risks depending on four different combinations of sweet food intake and total DSQ score (Table 5). Compared with the participants who consumed sweets less than the median of 1.71 daily servings and had total DSQ scores higher than the median of 4.43, those with lower sweet consumption but lower DSQ scores had increased ACS risks with an OR of 8.64 (95% CI 2.06–43.77). For a group of participants who had higher DSQ scores but consumed larger quantities of sweets, the OR for ACS was 14.99 (95% CI 3.42–85.02). A combination of higher sweet consumption and lower total DSQ score further increased the risk by an OR of 20.72 (95% CI 5.10–111.64).

**Table 5:** Odd ratios of ACS associated with the combinations of select lifestyle factors.

	***n***	**Sweet intake**	**Total DSQ score**	**OR (95% Cl)**	

				**Model 1** ^a^	**Model 2** ^b^
Sweets (low) × DSQ (high)	35	0.50 ± 0.55	5.08 ± 0.58	1	1
Sweets (low) × DSQ (low)	21	0.79 ± 0.61	3.61 ± 0.75	8.19 (2.05–39.65)^**^	8.64 (2.06–43.77)^**^
Sweets (high) × DSQ (high)	23	3.71 ± 1.48	5.02 ± 0.58	14.58 (3.53–77.15)^***^	14.99 (3.42–85.02)^***^
Sweets (high) × DSQ (low)	35	3.76 ± 2.29	3.67 ± 0.74	26.96 (6.50–149.67)^***^	20.72 (5.10–111.64)^***^

Values are presented as means ± SDs. *n* = 114. The odds ratios were determined using logistic regression model with adjustment for sex, age, and smoking. High sweet intake was defined as daily sweet intake more than the median of 1.71 daily servings, and high DSQ score as a total score higher than the median score of 4.43.

CI, confidence interval; OR, odds ratio.

^**^p < 0.01.

^***^p < 0.001.

^a^ORs for developing ACS were determined using generalized linear models with adjustment for sex, age, and smoking.

^b^ORs for developing ACS were determined using generalized linear models with adjustment for sex, age, smoking, and total energy intake.

## Discussion

The importance of maintaining healthy lifestyle choices has long been acknowledged in the prevention of CAD (28). However, integrative approaches to managing lifestyle components for CAD prevention and treatment are still limited in the field. In the current study, we identified the most distinguishing nutritional and psychological factors in the lifestyle of Korean ACS patients, which can be useful to understand lifestyle-associated risks in this population. We found that a dietary habit of consuming sweet foods that are high in added sugars and a lower level of overall life satisfaction were most significantly associated with increased risks of developing ACS individually and synergistically. Notably, as for psychological status, the measure of psychological wellbeing, i.e., life satisfaction, is better associated with the risk of ACS rather than that of psychological maladjustments in our study population.

The most noticeable characteristics in the diet of ACS patients, compared with that of the controls, were a high intake of sweets (>1.52 servings/day), which included added sugars in coffee/tea and several dessert foods. It was identified as a significant independent risk factor for ACS (OR 4.57). Intriguingly, the daily consumption of sugars added in coffee mixes was 2.36 servings out of 2.65 servings of overall sweet intake (~90%) in the ACS patients, which was almost three times higher than that of the controls. The consumption of added sugars has drawn great attention due to its exponential increases over the past few decades in many countries along with the obesity epidemic worldwide (29). According to a recent systematic analysis of the GBD study (6), the consumption of SSB was far higher than the optimal intake, especially in North and Latin America, Europe, and high-income Asia Pacific areas. In Western populations, the influences of consuming high amounts of added sugars or SSB on the risk of obesity/metabolic syndrome (30–32), type 2 diabetes (31, 33), hypertension (34, 35), and CAD (32, 36) have been well-established. Similar trends have been reported in Korean adults: the consumption of SSB is associated with a higher risk of obesity/metabolic syndrome (37, 38), hypertension (39), and CAD (40). While SSB has been identified to account for the largest proportion of added sugar intake in the Korean diet (41), the main source of SSB that drives the association with CAD risk remains relatively unstudied. It has been reported that instant coffee was the top source of sugar intake from SSB for Korean adults aged over 30 years but soft drinks for those under 30 years of age (42). This may be attributed to a distinctive coffee consumption pattern observed among Koreans, particularly in middle-aged or older generations. They prefer instant coffee mixes that come pre-packaged with sugar and non-dairy creamer, contrary to the increasing consumption of brewed black coffee among younger adults (43). Increased sugar intake, coupled with inadequate physical activity, may have contributed to the development of metabolic syndrome or type 2 diabetes in the ACS group, as indicated in their higher prevalence of diabetes (30 vs. 4%) and metabolic syndrome (33 vs. 4%) than the control group. This is in line with the increased risk of metabolic syndrome observed in Korean adults who have a high intake of coffee mixes (at least three times per day) (44). Taken together, our data suggest that high sweet food consumption could contribute significantly to the development of ACS probably, at least in part, through metabolic syndrome or type 2 diabetes.

Eating fish and/or seafood, as good sources of very long-chain omega-3 fatty acids and protein, has long been considered protective against CVD risk, and therefore, dietary recommendations generally encourage consuming a variety of fish, preferably oily fish, at least two servings per week for CVD prevention (45, 46). Unexpectedly, in our study, ACS patients had a higher intake of fish or seafood than the controls, although fish intake was not significantly associated with ACS risk. One thing to be considered is that the favorable associations reported between fish intake and CVD risk factors, such as dyslipidemia or high blood pressure among Korean adults, were specifically for oily fish or omega-3 fatty acid intake (47, 48). Furthermore, recent studies revealed heterogeneity in the associations between fish intake and CVD risk which vary by geographic region or CVD history of individuals (49, 50). The lower CVD risk with high fish intake was observed in CVD patients or high-risk individuals but not in general populations without CVD. Moreover, globally, the trend is neutral in general except for China and Africa (50), which may be attributable to different types of fish consumed, cooking methods, mercury levels, and environmental contaminants in fish.

In addition to diet, psychological factors are known to contribute to the development and prognosis of CAD (51, 52). In this study, we examined the association between psychological health and incident ACS by assessing both psychological maladjustments, including depressive symptoms and perceived stress, and psychological wellbeing such as life satisfaction. The ACS patients had higher levels of depressive symptoms with lower life satisfaction than the controls. The total DSQ score, which represents overall satisfaction across eight life domains, was inversely associated with ACS risk when adjusted for sex, age, and smoking status. This is consistent with a previous study that showed life satisfaction score as a predictor of physical health outcomes including mortality (53). As psychological health is now denoted as a state of complete wellbeing in various aspects (54), the measures of psychological wellbeing should also be included in the prevention and care of patients with CAD. Nonetheless, while the detrimental effects of psychological maladjustments, such as depression (55) and stress (52), on CAD risk have traditionally been examined, studies on associations between psychological wellbeing and cardiovascular health remain limited. In this context, our findings that life domain satisfaction, rather than depressive symptoms or perceived stress levels, is inversely associated with ACS risk add to the evidence on the beneficial role of maintaining psychological wellbeing in cardiovascular health, especially for individuals with suboptimal levels of psychological maladjustment.

The influence of psychological status on cardiovascular health is beyond its independent effect because psychological maladjustment tends to be accompanied by other unfavorable lifestyle changes, for example, smoking and an unhealthy diet, thereby increasing the risk of CAD (52). We also observed a modest but significant inverse correlation between overall life satisfaction and sweet intake. Reversely, the impact of high sugar intake on increasing the risk of psychological disorders has also been proposed with plausible biological mechanisms (56). Sugar overconsumption can alter neural pathways that are involved in addiction, stress, and depression (56), and several hormone levels that have the potential to affect mood states (57). Based on these links reported between psychological health and diet, we further assessed their interactive association with the risk of developing ACS using a subgroup analysis. Participants with a combination of daily sweet intake higher than 1.71 servings and total DSQ scores lower than 4.43 had a higher ACS risk with an OR of 20.72 compared with those with lower sweet intake and higher DSQ scores. Our data suggest a synergistically interactive role of these lifestyle factors in developing CAD, emphasizing a demand for a more comprehensive approach to secondary prevention.

We acknowledge that this study has several limitations. Our patient data was collected cross-sectionally after the incidence of ACS events. Though we intentionally asked the patients to recall their dietary habits and psychological status for a period of 1 year and 2–4 weeks, respectively, preceding the ACS events, this might have introduced some bias in their answers. In addition, the control group was not perfectly matched with the ACS patients in terms of sex and age due to practical challenges in recruitment. To account for these differences between the groups, we have taken sex, age, and other potential variations as covariates in all our analyses. In addition, because the FFQ used in this study was not strictly designed to assess the amount of added sugars or SSB consumed, we used sweet intake as a proxy variable. Nonetheless, our study provides useful information on the main components that best represent the dietary and psychological status of Korean ACS patients and their potential interaction, which could lay the groundwork for secondary prevention program development. To the best of our knowledge, this is the first study that identifies both dietary and psychological factors associated with CAD risk, especially in Korean adults, and further suggests their synergistic role. Furthermore, the strength of the present study is that psychological wellbeing factors and psychological maladjustment factors were assessed together.

## Conclusion

Our data show that high sweet food intake and low life satisfaction can act as risk factors for ACS through a potential synergistic interaction. This urges improvements in current cardiac rehabilitation programs to deal with comprehensive core components including patient-centered diet counseling and psychological factors pertaining to a specific population for greater effectiveness.

## Data availability statement

The raw data supporting the conclusions of this article will be made available by the authors, without undue reservation.

## Ethics statement

The studies involving human participants were reviewed and approved by the Institutional Review Board of Ewha Womans University Mokdong Hospital. The patients/participants provided their written informed consent to participate in this study.

## Author contributions

I-MC, K-MC, and SNH designed the research. JSo, JSeo, and HC conducted the data collection. JSo, JSeo, BK, K-MC, SNH, and I-MC conducted the sample and data analyses. JSo and KC wrote the manuscript. JSo, K-MC, SNH, and I-MC had primary responsibility for the final content. All authors have read and approved the final manuscript.

## References

[1] Naghavi M Abajobir AA Abbafati C Abbas KM Abd-Allah F Abera SF. Global, regional, and national age-sex specific mortality for 264 causes of death, 1980-2016: a systematic analysis for the Global Burden of Disease Study 2016. Lancet. (2017) 390:1151–210. 10.1016/S0140-6736(17)32152-9 28919116 PMC5605883

[2] Ford ES Ajani UA Croft JB Critchley JA Labarthe DR Kottke TE. Explaining the decrease in U.S. deaths from coronary disease, 1980-2000. N Engl J Med. (2007) 356:2388–98. 10.1056/NEJMsa053935 17554120

[3] Grundy SM Stone NJ Bailey AL Beam C Birtcher KK Blumenthal RS. 2018 AHA/ACC/AACVPR/AAPA/ABC/ACPM/ADA/AGS/APhA/ASPC/NLA/PCNA Guideline on the management of blood cholesterol: a report of the American College of Cardiology/American Heart Association Task Force on clinical practice guidelines. Circulation. (2019) 139:E1082–143. 10.1161/CIR.0000000000000624 30586774 PMC7403606

[4] Kelsey MD Nelson AJ Green JB Granger CB Peterson ED McGuire DK. Guidelines for cardiovascular risk reduction in patients with type 2 diabetes: JACC guideline comparison. J Am Coll Cardiol. (2022) 79:1849–57. 10.1016/j.jacc.2022.02.046 35512864 PMC8972581

[5] Supervia M Turk-Adawi K Lopez-Jimenez F Pesah E Ding R Britto RR. Nature of cardiac rehabilitation around the globe. EClinicalMedicine. (2019) 13:46. 10.1016/j.gheart.2018.09.026 31517262 PMC6733999

[6] Afshin A Sur PJ Fay KA Cornaby L Ferrara G Salama JS. Health effects of dietary risks in 195 countries, 1990–2017: a systematic analysis for the Global Burden of Disease Study 2017. Lancet. (2019) 393:1958–72. 10.1016/S0140-6736(19)30041-8 30954305 PMC6899507

[7] Appel LJ Moore TJ Obarzanek E Vollmer WM Svetkey LP Sacks FM. A clinical trial of the effects of dietary patterns on blood pressure. DASH collaborative research group. N Engl J Med. (1997) 336:1117–24. 10.1056/NEJM199704173361601 9099655

[8] Lichtenstein AH Appel LJ Lichtenstein AH Appel LJ Vadiveloo M Hu FB. 2021 Dietary guidance to improve cardiovascular health: a scientific statement from the American Heart Association. Circulation. (2021) 144:472–87. 10.1161/CIR.0000000000001031 34724806

[9] de Abreu D Guessous I Vaucher J Preisig M Waeber G Vollenweider P. Low compliance with dietary recommendations for food intake among adults. Clin Nutr. (2013) 32:783–8. 10.1016/j.clnu.2012.11.022 23260749

[10] Rehm CD Peñalvo JL Afshin A Mozaffarian D. Dietary intake among US adults, 1999-2012. JAMA. (2016) 315:2542–53. 10.1001/jama.2016.7491 27327801 PMC6287627

[11] Hendrie GA Golley RK Noakes M. Compliance with dietary guidelines varies by weight status: a cross-sectional study of Australian adults. Nutrients. (2018) 10:197. 10.3390/nu10020197 29439463 PMC5852773

[12] Pivato CA Chandiramani R Petrovic M Nicolas J Spirito A Cao D. Depression and ischemic heart disease. Int J Cardiol. (2022) 364:9–15. 10.1016/j.ijcard.2022.05.056 35643217

[13] De Hert M Detraux J Vancampfort D. The intriguing relationship between coronary heart disease and mental disorders. Dialogues Clin Neurosci. (2018) 20:31–40. 10.31887/DCNS.2018.20.1/mdehert 29946209 PMC6016051

[14] Trudel-Fitzgerald C Reduron LR Kawachi I Kubzansky LD. Specificity in associations of anger frequency and expression with different causes of mortality over 20 years. Psychosom Med. (2021) 83:402–9. 10.1097/PSY.0000000000000948 33901055 PMC8178222

[15] Sara JD Prasad M Eleid MF Zhang M Jay Widmer R Lerman A. Association between work-related stress and coronary heart disease: a review of prospective studies through the job strain, effort-reward balance, and organizational justice models. J Am Heart Assoc. (2018) 7:e008073. 10.1161/JAHA.117.008073 29703810 PMC6015274

[16] World Health Organization. World Health Report 1998: Life in the 21st Century, A Vision for All. Geneva: WHO (1998), p. 39.

[17] Francis T Kabboul N Rac V Mitsakakis N Pechlivanoglou P Bielecki J. The effect of cardiac rehabilitation on health-related quality of life in patients with coronary artery disease: a meta-analysis. Can J Cardiol. (2019) 35:352–64. 10.1016/j.cjca.2018.11.013 30825955

[18] De Menezes Caceres V Stocks N Adams R Haag DG Peres KG Peres MA. Physical activity moderates the deleterious relationship between cardiovascular disease, or its risk factors, and quality of life: findings from two population-based cohort studies in Southern Brazil and South Australia. PLoS ONE. (2018) 13:e0198769 10.1371/journal.pone.0198769 29879229 PMC5991645

[19] Ryff CD Dienberg Love G Urry HL Muller D Rosenkranz MA Friedman EM. Psychological well-being and ill-being: do they have distinct or mirrored biological correlates? Psychother Psychosom. (2006) 75:85–95. 10.1159/000090892 16508343

[20] Huppert FA Whittington JE. Evidence for the independence of positive and negative well-being: implications for quality of life assessment. Br J Health Psychol. (2003) 8:107–22. 10.1348/135910703762879246 12643820

[21] van Halewijn G Deckers J Tay HY van Domburg R Kotseva K Wood D. Lessons from contemporary trials of cardiovascular prevention and rehabilitation: a systematic review and meta-analysis. Int J Cardiol. (2017) 232:294–303. 10.1016/j.ijcard.2016.12.125 28094128

[22] Ahn Y Lee J-E Paik H-Y Lee H-K Jo I Kimm K. Development of a semi-quantitative food frequency questionnaire based on dietary data from the Korea National Health and Nutrition Examination Survey. Nutr Sci. (2003) 6:173–84.

[23] Ahn Y Kwon E Shim JE Park MK Joo Y Kimm K. Validation and reproducibility of food frequency questionnaire for Korean genome epidemiologic study. Eur J Clin Nutr. (2007) 61:1435–41. 10.1038/sj.ejcn.1602657 17299477

[24] Kroenke K Spitzer RL Williams JBW. The PHQ-9: validity of a brief depression severity measure. J Gen Intern Med. (2001) 16:606–13. 10.1046/j.1525-1497.2001.016009606.x 11556941 PMC1495268

[25] Cohen S Kamarck T Mermelstein R. A global measure of perceived stress. J Health Soc Behav. (1983) 24:385–96. 10.2307/2136404 6668417

[26] Boehm JK Peterson C Kivimaki M Kubzansky LD. Heart health when life is satisfying: evidence from the Whitehall II cohort study. Eur Heart J. (2011) 32:2672–7. 10.1093/eurheartj/ehr203 21727096 PMC3205478

[27] R Core Team. R: A Language Environment for Statistical Computing. Vienna: R Foundation for Statistical Computing (2021). Available online at: https://www.Rproject.org/

[28] German CA Baum SJ Ferdinand KC Gulati M Polonsky TS Toth PP. Defining preventive cardiology: a clinical practice statement from the American Society for Preventive Cardiology. Am J Prev Cardiol. (2022) 12:100432. 10.1016/j.ajpc.2022.100432 36425534 PMC9679464

[29] World Cancer Research Fund International. Curbing Global Sugar Consumption: Effective Food Policy Actions to Help Promote Healthy Diets and Tackle Obesity. London: World Cancer Research Fund International (2015).

[30] Ebbeling CB Feldman HA Osganian SK Chomitz VR Ellenbogen SJ Ludwig DS. Effects of decreasing sugar-sweetened beverage consumption on body weight in adolescents: a randomized, controlled pilot study. Pediatrics. (2006) 117:673–80. 10.1542/peds.2005-0983 16510646

[31] Hu FB Malik VS. Sugar-sweetened beverages and risk of obesity and type 2 diabetes: epidemiologic evidence. Physiol Behav. (2010) 100:47–54. 10.1016/j.physbeh.2010.01.036 20138901 PMC2862460

[32] Dhingra R Sullivan L Jacques PF Wang TJ Fox CS Meigs JB. Soft drink consumption and risk of developing cardiometabolic risk factors and the metabolic syndrome in middle-aged adults in the community. Circulation. (2007) 116:480–8. 10.1161/CIRCULATIONAHA.107.689935 17646581

[33] Schulze MB Manson JAE Ludwig DS Colditz GA Stampfer MJ Willett WC. Sugar-sweetened beverages, weight gain, and incidence of type 2 diabetes in young and middle-aged women. JAMA. (2004) 292:927–34. 10.1001/jama.292.8.927 15328324

[34] Chen L Caballero B Mitchell DC Loria C Lin PH Champagne CM. Reducing consumption of sugar-sweetened beverages is associated with reduced blood pressure: a prospective study among United States adults. Circulation. (2010) 121:2398–406. 10.1161/CIRCULATIONAHA.109.911164 20497980 PMC2892032

[35] Sayon-Orea C Martinez-Gonzalez MA Gea A Alonso A Pimenta AM Bes-Rastrollo M. Baseline consumption and changes in sugar-sweetened beverage consumption and the incidence of hypertension: the SUN project. Clin Nutr. (2015) 34:1133–40. 10.1016/j.clnu.2014.11.010 25481680

[36] Yang Q Zhang Z Gregg EW Flanders WD Merritt R Hu FB. Added sugar intake and cardiovascular diseases mortality among US adults. JAMA Intern Med. (2014) 174:516–24. 10.1001/jamainternmed.2013.13563 24493081 PMC10910551

[37] Kang Y Kim J. Soft drink consumption is associated with increased incidence of the metabolic syndrome only in women. Br J Nutr. (2017) 117:315–24. 10.1017/S0007114517000046 28166856

[38] Shin S Kim SA Ha J Lim K. Sugar-sweetened beverage consumption in relation to obesity and metabolic syndrome among Korean adults: a cross-sectional study from the 2012–2016 Korean National Health and Nutrition Examination Survey (KNHANES). Nutrients. (2018) 10:1467. 10.3390/nu10101467 30304842 PMC6213560

[39] Kwak JH Jo G Chung HK Shin MJ. Association between sugar-sweetened beverage consumption and incident hypertension in Korean adults: a prospective study. Eur J Nutr. (2019) 58:1009–17. 10.1007/s00394-018-1617-1 29372311

[40] Kim SO Bae EM Lee YN Son JS. Association between consumption of sugar-sweetened beverages and risk of cardiovascular disease in Korean men: analysis based on the Korea National Health and Nutrition Examination Survey 2014-2016. Korean J Fam Med. (2021) 42:212–8. 10.4082/kjfm.20.0114 34038989 PMC8164932

[41] Shim J-S. Ultra-processed foods and total sugars intake in Korea: evidence from the Korea National Health and Nutrition Examination Survey 2016–2018. Nutr Res Pract. (2022) 16:476–88. 10.4162/nrp.2022.16.4.476 35919288 PMC9314195

[42] Lee HS Kwon SO Yon M Kim D Lee JY Nam J. Dietary total sugar intake of Koreans: based on the Korea National Health and Nutrition Examination Survey (KNHANES), 2008-2011. J Nutr Health. (2014) 47:268–76. 10.4163/jnh.2014.47.4.268

[43] Je Y Jeong S Park T. Coffee consumption patterns in Korean adults: the Korean National Health and Nutrition Examination Survey (2001-2011). Asia Pac J Clin Nutr. (2014) 23:691–702. 10.6133/apjcn.2014.23.4.11 25516328

[44] Kim HJ Cho S Jacobs DR Park K. Instant coffee consumption may be associated with higher risk of metabolic syndrome in Korean adults. Diabetes Res Clin Pract. (2014) 106:145–53. 10.1016/j.diabres.2014.07.007 25112922

[45] Arnett DK Blumenthal RS Albert MA Buroker AB Goldberger ZD Hahn EJ. 2019 ACC/AHA guideline on the primary prevention of cardiovascular disease: a report of the American College of Cardiology/American Heart Association Task Force on clinical practice guidelines. Circulation. (2019) 140:e596–646. 10.1161/CIR.0000000000000725 30879355 PMC7734661

[46] U.S. Department of Agriculture an d U.S. Department of Health and Human Services. Dietary Guidelines for Americans, 2020-2025, 9th ed. (2020). Available online at: https://www.dietaryguidelines.gov/ (accessed July 23, 2023).

[47] Kim SA Lee JK Kang D Shin S. Oily fish consumption and the risk of dyslipidemia in Korean adults: a prospective cohort study based on the health examinees gem (HEXA-G) study. Nutrients. (2019) 11:2506. 10.3390/nu11102506 31627478 PMC6835780

[48] Kim H Park S Yang H Choi YJ Huh KB Chang N. Association between fish and shellfish, and omega-3 PUFAs intake and CVD risk factors in middle-aged female patients with type 2 diabetes. Nutr Res Pract. (2015) 9:496–502. 10.4162/nrp.2015.9.5.496 26425279 PMC4575962

[49] Wu JHY Micha R Imamura F Pan A Biggs ML Ajaz O. Omega-3 fatty acids and incident type 2 diabetes: a systematic review and meta-analysis. Br J Nutr. (2012) 107(Suppl 2):S214–27. 10.1017/S0007114512001602 22591895 PMC3744862

[50] Mohan D Mente A Dehghan M Rangarajan S O'Donnell M Hu W. Associations of fish consumption with risk of cardiovascular disease and mortality among individuals with or without vascular disease from 58 countries. JAMA Intern Med. (2021) 181:631–49. 10.1001/jamainternmed.2021.0036 33683310 PMC7941252

[51] Rozanski A Blumenthal JA Kaplan J. Impact of psychological factors on the pathogenesis of cardiovascular disease and implications for therapy. Circulation. (1999) 99:2192–217. 10.1161/01.CIR.99.16.2192 10217662

[52] Kivimäki M Steptoe A. Effects of stress on the development and progression of cardiovascular disease. Nat Rev Cardiol. (2018) 15:215–29. 10.1038/nrcardio.2017.189 29213140

[53] Koivumaa-Honkanen H Honkanen R Viinamäki H Heikkilä K Kaprio J Koskenvuo M. Self-reported life satisfaction and 20-year mortality in healthy Finnish adults. Am J Epidemiol. (2000) 152:983–91. 10.1093/aje/152.10.983 11092440

[54] Levav I Rutz W. The WHO world health report 2001 new understanding–new hope. Isr J Psychiatry Relat Sci. (2002) 39:50–6. 12013710

[55] Carney RM Freedland KE. Depression and coronary heart disease. Nat Rev Cardiol. (2017) 14:145–55. 10.1038/nrcardio.2016.181 27853162

[56] Jacques A Chaaya N Beecher K Ali SA Belmer A Bartlett S. The impact of sugar consumption on stress driven, emotional and addictive behaviors. Neurosci Biobehav Rev. (2019) 103:178–99. 10.1016/j.neubiorev.2019.05.021 31125634

[57] Sen S Duman R Sanacora G. Serum brain-derived neurotrophic factor, depression, and antidepressant medications: meta-analyses and implications. Biol Psychiatry. (2008) 64:527–32. 10.1016/j.biopsych.2008.05.005 18571629 PMC2597158

