# Supplementary material for: High intake of sweet foods and low life satisfaction can act as risk factors for acute coronary syndrome through synergistic interaction
Source: Front Nutr. 2023 Aug 7;10:1221916. doi: 10.3389/fnut.2023.1221916 (PMC10440423; doi:10.3389/fnut.2023.1221916)
Supplement: Supplementary file 1 [file Data_Sheet_1.docx]

**Supplementary table legends**

**Supplementary Table 1.** Grouping of FFQ food items.

**Supplementary Table 2.** Socioeconomic status of study participants.

**Supplementary Table 3.** Medication use of ACS patients.

**Supplementary Table 4.** Nutrient and food group intakes in ACS patients and controls.

**Supplementary Table 5.** Intakes of individual food items composing the food groups sweets and fish/seafood in ACS patients and controls.

| **Supplementary Table 1.** Grouping of FFQ food items. | | | |
| --- | --- | --- | --- |
| Food groups | Items | Food groups | Items |
| Rice and grains | White rice |  | Dog meat |
|  | White rice with soybeans |  | Korean style meat soup |
|  | Multi-grain rice |  | Dumpling |
|  | Half white rice/half white rice with soybeans |  | Fried/steamed chicken |
|  | Half white rice/half multi-grain rice | Fish and seafood | Sashimi |
|  | Rice cake (made with white rice) |  | Mackerel/saury |
|  | Rice cake (made with other types of grain) |  | Hairtail |
|  | Powdered mixed grains |  | Eel |
|  | Cereals |  | Croaker/flatfish/sea bream |
| Starch and  starch vegetables | Muk (a jelly-like food made with nut starch) |  | Pollack |
|  | Potato |  | Squid/dried squid/octopus |
|  | Sweet potato |  | Anchovy |
|  | Korean vermicelli  (made with sweet potato starch) |  | Tuna/canned tuna |
|  |  |  | Clam/conch |
| Noodles | Wheat noodle/udon |  | Oyster |
|  | Chinese style noodles  (with stir-fried bean paste or spicy soup) |  | Crab/salted crab |
|  |  |  | Shrimp |
|  | Cold noodles  (made with vermicelli or buckwheat) |  | Fish cake/crab meat |
|  |  | Salted fish | Salted fish |
| Instant ramen | Instant ramen | Eggs | Eggs/quail eggs |
| Meat and poultry | Pork belly | Legumes | Black bean |
|  | Grilled/fried pork |  | Korean miso |
|  | Braised pork |  | Tofu |
|  | Processed meat |  | Soy milk |
|  | Offal/organ meat/tripe | Nuts | Peanut/almond/pine nut |
|  | Beef steak |  |  |
| Fruits | Strawberry | Salted vegetables | Cabbage Kimchi |
|  | Melon (Korean melon/melon) |  | White radish Kimchi |
|  | Watermelon |  | Watery Kimchi with cabbage/radish |
|  | Peach/plum |  | Other types of Kimchi |
|  | Banana |  | Pickled vegetables |
|  | Persimmon/dried persimmon | Seaweeds | Seaweeds |
|  | Mandarin | Dairy | Milk |
|  | Pear |  | Yogurt |
|  | Apple |  | Ice cream |
|  | Orange/orange juice |  | Cheese |
|  | Grape/grape juice | Sweets | Candy/chocolates |
|  | Tomato/tomato juice/ketchup |  | Table sugar (added sugar in coffee/tea) |
| Vegetables | White radish/daikon |  | Cake/moon pie (or whoopie pie) |
|  | Napa cabbage |  | Bread with sweet red bean |
|  | Spinach |  | Soda |
|  | Lettuce | Fast foods | Pizza/hamburger |
| Others | Jam/honey/margarine |  |  |
|  | Coffee cream |  |  |
|  | Sandwich bread |  |  |
|  | Other type of bread |  |  |
|  | Cookies/crackers/snack |  |  |
|  | Coffee (black) |  |  |
|  | Other types of beverages (e.g. tea) |  |  |

| **Supplementary Table 2.** Socioeconomic status of study participants. | | | |
| --- | --- | --- | --- |
|  | ACS patients  (n=92) | Controls  (n=69) | *P*^1^ |
| Education (%) |  |  | 0.26 |
| High school or less | 48 (52.2) | 25 (36.2) |  |
| College graduate or more | 34 (37.0) | 36 (52.2) |  |
| No response | 10 (10.9) | 8 (11.6) |  |
|  |  |  |  |
| Household income (%) |  |  | **< 0.001** |
| Upper 25% | 9 (9.8) | 25 (36.2) |  |
| Middle 50% | 46 (50.0) | 33 (47.8) |  |
| Lower 25% | 25 (27.2) | 3 (4.3) |  |
| No response | 12 (13.0) | 8 (11.6) |  |
|  |  |  |  |
| Marital status (%) |  |  | 0.76 |
| Married | 61 (66.3) | 59 (85.5) |  |
| Divorced, separated, widowed | 14 (15.2) | 1 (1.4) |  |
| Never married | 6 (6.5) | 3 (4.3) |  |
| No response | 11 (12.0) | 6 (8.7) |  |

^1^*P* values for the comparison between ACS patients and controls were determined using generalized linear models with adjustment for sex and age.

| **Supplementary Table 3.** Medication use of ACS patients. | |
| --- | --- |
| ACE inhibitor (%) | 63 (68.5) |
| ARB (%) | 2 (2.2) |
| Beta blocker (%) | 37 (40.2) |
| Calcium channel blocker (%) | 10 (10.9) |
| Statin (%) | 64 (69.6) |
| Antiplatelets (%) | 91 (98.9) |

ACE, angiotensin converting enzyme; ARB, angiotensin II type 1 receptor inhibitor. n=92.

| **Supplementary Table 4.** Nutrient and food group intakes in ACS patients and controls. | | | |
| --- | --- | --- | --- |
| Variable | ACS  (n=91) | Control  (n=61) | *P* |
| *a) Energy intakes*^†^, *kcal* | 2066 ± 508 | 1855 ± 560 | **0.02** |
|  |  |  |  |
| *b) Nutrient intakes* |  |  |  |
| Macronutrients |  |  |  |
| Carbohydrate, g | 373 ± 90 | 336 ± 104 | 0.74 |
| Dietary fiber, g | 20 ± 6 | 19 ± 9 | 0.15 |
| Fat, g | 38 ± 19 | 34 ± 16 | 0.77 |
| Cholesterol, mg | 241 ± 129 | 206 ± 140 | 0.37 |
| SFA, g | 9.6 ± 5.5 | 8.4 ± 4.7 | 0.47 |
| MUFA, g | 6.2 ± 9.0 | 9.0 ± 5.1 | 0.53 |
| PUFA, g | 5.2 ± 2.9 | 4.2 ± 1.9 | 0.22 |
| Protein, g | 77 ± 24 | 68 ± 23 | 0.49 |
| Fat-soluble vitamins |  |  |  |
| Vitamin A, μg RAE | 568 ± 270 | 605 ± 366 | 0.17 |
| Vitamin D, μg | 4.3 ± 3.5 | 2.9 ± 1.8 | **0.04** |
| Vitamin E, mg α-TE | 9.4 ± 3.7 | 8.2 ± 3.2 | 0.82 |
| Vitamin K, μg | 184 ± 129 | 174 ± 147 | 0.78 |
| Water-soluble vitamins |  |  |  |
| Vitamin C, mg | 85 ± 44 | 93 ± 61 | 0.16 |
| Niacin, mg NE | 17 ± 6 | 15 ± 6 | 0.81 |
| Vitamin B6, mg | 1.6 ± 0.4 | 1.5 ± 0.6 | 0.49 |
| Folate, μg DFE | 492 ± 162 | 463 ± 203 | 0.82 |
| Vitamin B12, μg | 7.7 ± 4.3 | 5.6 ± 3.3 | **0.03** |
| Minerals |  |  |  |
| Calcium, mg | 471 ± 183 | 435 ± 221 | 0.81 |
| Phosphorus, mg | 1069 ± 316 | 940 ± 344 | 0.36 |
| Sodium, mg | 2927 ± 1263 | 2550 ± 1400 | 0.50 |
| Iron, mg | 14 ± 5 | 13 ± 5 | 0.62 |
| Selenium, μg | 115 ± 34 | 97 ± 32 | **0.02** |
|  |  |  |  |
| *c) Food group intakes, serving*^††^ |  |  |  |
| Rice and grains | 2.85 ± 0.82 | 2.47 ± 0.96 | 0.55 |
| Starch and starchy vegetables | 0.55 ± 0.73 | 0.42 ± 0.45 | 0.31 |
| Noodles | 0.21 ± 0.25 | 0.24 ± 0.32 | **0.04** |
| Instant ramen | 0.15 ± 0.19 | 0.12 ± 0.20 | 0.53 |
| Meat and poultry | 0.31 ± 0.33 | 0.30 ± 0.44 | 0.84 |
| Fish and seafood | 1.82 ± 1.32 | 1.13 ± 0.73 | **0.008** |
| Salted fish | 0.06 ± 0.12 | 0.04 ± 0.06 | 0.25 |
| Eggs | 0.31 ± 0.33 | 0.30 ± 0.44 | 0.99 |
| Legumes | 0.94 ± 1.03 | 0.86 ± 0.70 | 0.52 |
| Nuts | 0.15 ± 0.29 | 0.15 ± 0.24 | 0.84 |
| Fruits | 1.32 ± 1.32 | 1.69 ± 1.89 | **<0.05** |
| Vegetables | 3.25 ± 2.33 | 3.44 ± 3.01 | 0.61 |
| Salted vegetables | 2.96 ± 1.97 | 2.58 ± 1.91 | 0.52 |
| Seaweeds | 0.61 ± 0.44 | 0.53 ± 0.61 | 0.57 |
| Dairy | 0.77 ± 0.85 | 0.79 ± 1.02 | 0.43 |
| Sweets | 2.65 ± 1.98 | 1.16 ± 2.14 | **<0.001** |
| Fast foods | 0.05 ± 0.13 | 0.03 ± 0.04 | 0.24 |

Values are means ± SDs. n=152. *P* values for the comparison between ACS and control groups were determined using generalized linear models with adjustment for sex, age, and total energy intake.

^†^*P* value adjusted for sex and age.

^††^ See Supplementary Table 1 for food grouping details.

| **Supplementary Table 5.** Intakes of individual food items composing the food groups sweets and fish/seafood in ACS patients and controls. | | | |
| --- | --- | --- | --- |
| Variable | ACS  (n=91) | Control  (n=61) | *P* |
| Sweets |  |  |  |
| Candy/chocolates | 0.05 ± 0.09 | 0.05 ± 0.08 | 0.88 |
| Table sugar (e.g. added sugar in coffee/tea) | 2.36 ± 1.96 | 0.80 ± 1.14 | **<0.001** |
| Cake/moon pie (or whoopie pie) | 0.02 ± 0.06 | 0.03 ± 0.04 | 0.09 |
| Bread with sweet red bean | 0.06 ± 0.12 | 0.03 ± 0.05 | 0.20 |
| Soda | 0.15 ± 0.42 | 0.25 ± 1.29 | 0.54 |
|  |  |  |  |
| Fish and seafood |  |  |  |
| Sashimi | 0.22 ± 0.52 | 0.09 ± 0.10 | 0.24 |
| Mackerel/saury | 0.27 ± 0.30 | 0.13 ± 0.13 | **0.003** |
| Hairtail | 0.09 ± 0.16 | 0.07 ± 0.08 | 0.62 |
| Eel | 0.03 ± 0.07 | 0.04 ± 0.07 | 0.42 |
| Croaker/flatfish/sea bream | 0.11 ± 0.15 | 0.09 ± 0.17 | 0.69 |
| Pollack | 0.11 ± 0.17 | 0.09 ± 0.14 | 0.67 |
| Squid/dried squid/octopus | 0.09 ± 0.18 | 0.06 ± 0.08 | 0.27 |
| Anchovy | 0.37 ± 0.48 | 0.25 ± 0.30 | 0.25 |
| Tuna/canned tuna | 0.04 ± 0.09 | 0.04 ± 0.05 | 0.93 |
| Clam/conch | 0.10 ± 0.32 | 0.06 ± 0.07 | 0.31 |
| Oyster | 0.07 ± 0.15 | 0.03 ± 0.05 | 0.29 |
| Crab/salted crab | 0.07 ± 0.13 | 0.03 ± 0.04 | 0.15 |
| Shrimp | 0.08 ± 0.14 | 0.08 ± 0.15 | 0.89 |
| Fish cake/crab meat | 0.16 ± 0.50 | 0.07 ± 0.10 | 0.14 |

Values are means ± SDs. n=152. *P* values for the comparison between ACS and control groups were determined using generalized linear models with adjustment for sex, age, and total energy intake.
